# Stable Ectopic Expression of *ST6GALNAC5* Induces Autocrine MET Activation and Anchorage-Independence in MDCK Cells

**DOI:** 10.1371/journal.pone.0148075

**Published:** 2016-02-05

**Authors:** Chia Chu, Donald P. Bottaro, Michael J. Betenbaugh, Joseph Shiloach

**Affiliations:** 1 Biotechnology Core Laboratory, National Institute of Diabetes and Digestive and Kidney Diseases, National Institutes of Health, Bethesda, Maryland, United States of America; 2 Department of Chemical and Biomolecular Engineering, Johns Hopkins University, Baltimore, Maryland, United States of America; 3 Urologic Oncology Branch, Center for Cancer Research, National Cancer Institute, National Institutes of Health, Bethesda, Maryland, United States of America; Hunter College of The City University of New York, UNITED STATES

## Abstract

The epithelial-mesenchymal transition (EMT) is a complex cancer progression that can boost the metastatic potential of transformed cells by inducing migration, loss of cell adhesion, and promoting proliferation under anchorage-independent conditions. A DNA microarray analysis was performed comparing parental anchorage-dependent MDCK cells and anchorage-independent MDCK cells that were engineered to express human *siat7e* (*ST6GALNAC5*). The comparison identified several genes involved in the EMT process that were differentially expressed between the anchorage-dependent and the anchorage-independent MDCK cell lines. The hepatocyte growth factor gene (*hgf*) was found to be over-expressed in the engineered MDCK-*siat7e* cells at both transcription and protein expression levels. Phosphorylation analysis of the MET receptor tyrosine kinase confirmed the activation of an autocrine loop of the HGF/ MET signaling pathway in the MDCK-*siat7e* cells. When MET activities were suppressed by using the small-molecular inhibitor drug PF-02341066 (Crizotinib), the anchorage-independent MDCK-*siat7e* cells reverted to the cellular morphology of the parental anchorage-dependent MDCK cells. These observations indicate that the MET receptor plays a central role in the growth properties of the MDCK cells and its phosphorylation status is likely dependent on sialylation. Further investigation of the downstream signaling targets in the MET network showed that the degree of MDCK cell adhesion correlated with secretion levels of a matrix metalloproteinase, MMP1, suggesting a role of metalloproteinases in the EMT process. These results demonstrate that in addition to its application in biotechnology processes, MDCK-*siat7e* may serve as a model cell for metastasis studies to decipher the sequence of events leading up to the activation of EMT.

## Introduction

Due to the labor-intensive nature of utilizing adherent mammalian cells for large-scale production of biologicals, a number of adherent cell lines have been adapted to grow in suspension [1]. The adaptation process is cumbersome, time-consuming, dependent on growth media, and does not always result in a stable suspension cell line. An alternative approach for developing suspension cell lines is genetic manipulation. Previous reports have demonstrated the effects of over-expressing anti-apoptotic genes such as Blc-2, p21CIP1, cyclins E and D1, survivin, and cMyc in transforming Chinese hamster ovary cells from surface attachment to suspension [2–6], and a similar effect when over-expressing *siat7e* in HeLa cells [7].

Madin Darby canine kidney (MDCK) cells, which are anchorage-dependent and efficient producers of several medically-relevant families of viruses, were converted to anchorage-independent cells by stable transfection with the human *siat7e* gene. A high *siat7e*-expressing MDCK sub-clone was isolated and characterized for its ability to proliferate under anchorage-independent growth conditions and for its tumorigenic activity [8,9]. The *siat7e* gene, encoding the sialyltransferase 7E enzyme, is not commonly expressed in epithelial cell lines such as MDCK [10,11]. So far, 20 different sialyltransferase enzymes have been identified, cloned, and characterized [12,13]. They are subcategorized into different families according to their substrate specificities and similarities in structural motifs. Correlations between cell surface sialylation and metastatic potentials have been documented [14–16], and changes in cellular adhesion behaviors have been reported in tumor cell lines with elevated amounts of surface sialic acid residues [17,18].

In addition to their application in virus isolation and propagation, MDCK cells have been routinely used as a model cell line for studying epithelial-mesenchymal transition, because the cells actively respond to stimulation by exogenous hepatocyte growth factor (HGF) treatment [19–22]. Epithelial-mesenchymal transition (EMT) is characterized by loss of cell-cell adhesion, changes in normal cellular morphology, and resistance to anoikis (apoptosis due to loss of surface attachment) [23–25]. Following the identification of HGF, it has been shown that transgenic expression of the human *hgf* cDNA in MDCK cells can promote anchorage-independent growth [26]. In vivo, HGF is commonly secreted by cells of mesenchymal origin and activates the auto-phosphorylation of the MET receptor tyrosine kinase expressed on the surface of epithelial cells, which, in turn, triggers cellular processes essential for embryonic development and wound healing [27]. Several signal transduction programs, such as the MAPK, STAT3, and PI3K pathways, have been connected with the activation of MET [28–31]. In transformed cell lines, however, the activation of the MET receptor can lead to increased invasive growth [27]. For this reason, a variety of MET inhibitor drugs, such as PF-02341066 [32,33], were developed by biopharmaceutical research labs as potential treatment regimens for cancer patients.

Molecular events leading to the activation of oncogenic pathways that occur during EMT have been intensively investigated for the purpose of discovering new drug targets for various oncology indications. These events are typically associated with the down-regulation of genes essential to cell-cell and cell-matrix adhesion. Recently, increased attention has been directed to the role of matrix metalloproteinase enzymes in metastatic transformation of cells [34–37]. Matrix metalloproteinases are either secreted or expressed as membrane-bound enzymes to degrade components in the extracellular matrix to free up bound cells [38]; this large family of enzymes can cleave all known components in the extracellular matrix. In a recent study, an unusually high mRNA level of *mmp1* was detected in *ras*-transformed MDCK cells [36], suggesting the possibility of its critical effector role in controlling the adhesion properties of the cells. In this report, we describe a series of experiments involving comprehensive gene expression profiling aimed at explaining the behavioral differences between the anchorage-dependent MDCK and the anchorage-independent MDCK-*siat7e* cells. Based on the collective data, we propose an outline of biochemical events that occur as a result of *siat7e* expression in the MDCK cells, and the manner in which MET activation contributes to the transformation of MDCK cells from anchorage-dependence to suspension growth.

## Materials and Methods

### 1. Cell lines, reagents, and cloning of human *mmp1* gene

Madin Darby canine kidney (MDCK) cells were purchased from American Type Culture Collection (ATCC, Cat. No. CCL-34). *Siat7e*-expressing MDCK cells were established as described previously [9]. MDCK and *siat7e*-expressing MDCK cells were subcultured in 37°C, 5% CO_2_ humid incubator using Minimal Essential Medium (MEM) containing Earl’s salts and L-glutamine (Invitrogen) and supplemented with 10% v/v fetal bovine serum (Invitrogen). MDCK cells were subcultured in 37°C, 5% CO_2_ humid incubator using Dulbecco’s Modified Eagle Medium (DMEM) containing L-glutamine and sodium pyruvate (Invitrogen) and supplemented with fetal bovine serum (Invitrogen) at 10% v/v final concentration. All cell lines were routinely passaged on 75-cm^2^ or 162-cm^2^ T-flasks (Corning). Only cells with less than 20 passages were used for this study.

MGC cDNA clone of human *mmp1* gene (pOTB7-*mmp1*) and expression vector pcDNA3.1(+) were purchased from Invitrogen. The pOTB7-*mmp1* vector was transformed in a competent E. coli BL21 strain (F^-^ *ompT hsdS*_*B*_ (r_B_^-^ m_B_^-^) *gal dcm*, Novagen) to eliminate *dcm* methylation on plasmid before digestion. The amplified vector pOTB7-MMP1 was digested using *StuI* and *XhoI* enzymes (New England Biolabs) at 37°C for 2 h to release the gene insert. The cDNA was then purified using the QIAquick gel extraction kit (Qiagen) after separation by 1% agarose TBE gel electrophoresis. The expression vector pcDNA3.1(+) was digested using *PmeI* and *XhoI* enzymes (New England Biolabs) at 37°C for 1 h. The linearized plasmid was purified using QIAquick nucleotide removal kit and treated with alkaline phosphatase (New England Biolabs) to avoid self-ligation. After phosphatase treatment, the plasmid was re-purified using a nucleotide removal kit. The gene insert and the linearized pcDNA3.1(+) backbone were ligated at 16°C overnight. The ligation reaction was transformed using a competent E. coli DH5α strain, and ampicillin-resistant colonies were screened by colony PCR using forward primer sequence, 5’–TGAGGGGAACCCTCGCTGGG–3’, and reverse primer sequence, 5’–GGCCGAGTTCATGAGCCGCA–3’.

MDCK cells (ATCC) were transfected with the pcDNA3.1(+) and pcDNA3.1(+)-*mmp1* plasmids using lipofectamine 2000 (Invitrogen) under manufacturer’s standard protocol. Briefly, cells were seeded at 2 x 10^5^ cells/well in a 24-well plate the day before transfection. On the day of transfection, 0.8 μg of plasmid DNA and 2 μL of lipofectamine reagent pre-incubated in OptiMEM I (Invitrogen) were added to each well. After 24 h, transfected cells were expanded to 10-cm^2^ tissue culture dishes and selected with 500 μg/mL geneticin (Gibco). Stable colonies were isolated and expanded for analysis of gene expression and cellular adhesion properties.

### 2. RNA preparation and transcriptome profiling using DNA microarrays

MDCK, low *siat7e*-expressing MDCK, and high *siat7e*-expressing MDCK cells were expanded to at least 1 x 10^7^ cells to generate RNA samples. During the mid-log phase growth phase (approximately 70–80% confluence), cells were washed and maintained in serum-free MEM for 24 h to synchronize the cell cycle phases in individual cells. Serum-supplemented media were replaced after 24 h and cells were harvested by trypsinization at 95% confluence. Detached cells were washed twice with cold 1x phosphate buffer saline (PBS) to remove traces of trypsin activity. Washed cell pellets were disrupted with TRIzol reagent (Invitrogen) for 5 min at room temperature. Disrupted samples were then homogenized using the QIAshredder columns (Qiagen). Homogenized samples after addition of molecular grade chloroform (Mallinkrodt Chemicals) were then centrifuged to separate the nucleic acid rich phase. The upper aqueous phase containing RNA were carefully aspirated and mixed with an equal volume of 70% ethanol. The processed samples were then purified using the RNeasy kit (Qiagen). Only samples with a 260/280 ratio (analyzed by Nanodrop) of 1.8 and greater were used the microarray study.

Samples were analyzed using the GeneChip Canine Genome 2.0 arrays (Affymetrix). The hybridization, washing, signal detection, and preliminary data processing were performed in the Microarray Core Facility at the National Institutes of Diabetes and Digestive and Kidneys Diseases. Raw data files were processed and analyzed using the Partek Pro software. All raw data file were first normalized using the RMA method. Normalized files were then compared using the ANOVA method to calculate p-values between each comparison. An abbreviated gene list was generated using a p-value cut-off of 0.01. This list was filtered using a fold-change cut-off of ±2 to produce the list of differentially expressed genes among the three cell lines. Genes with consistent expressions patterns as the expression level of *siat7e* and relevant functionalities were targeted for further analyses.

### 3. End-point PCR and real-time PCR confirmation of target genes

For confirmation studies, a separate aliquot of RNA samples submitted for DNA microarray analysis were used. All primer sequences were designed using BLAST-primer program from the NCBI website and are as follow: GAPDH forward primer 5’-AACATCATCCCTGCTTCCAC-3’, GAPDH reverse primer 5’-GACCACCTGGTCCTCAGTGT-3’, MMP1 forward primer 5’-GACACGGACACCCTGAATCT-3’, MMP1 reverse primer 5’-TACCTCTGCTCTCGGCAAAT-3’, HGF forward primer 5’-CCCTGGGAGTACTGTGCAAT-3’, HGF reverse primer 5’-ATGCTGGTGAGGATACTGGG-3’, CDH1 forward primer 5’-CAAGCGGCCTCTACAACTTC-3’, CDH1 reverse primer 5’-AACTGGGAAATGTGAGCACC-3’, DSG2 forward primer 5’-CCACCTGAAGACAAGGTGGT-3’, DSG2 reverse primer 5’-GTCTTTGATGAGGGCAGAGC-3’, CLDN7 forward primer 5’-AAGTGTACCAACTGTGGGGG-3’, CLDN7 reverse primer 5’-GGGGTTGTAGAAGTCCGTGA-3’, EPCAM forward primer 5’-CAAAGTCTGGGAGAAGAGCG-3’, and EPCAM reverse primer 5’-AGCAGTGTTCACACACCAGC-3’. End-point PCR analysis was performed using SuperScript one-step RT-PCR kits (Invitrogen) following vendor’s protocols. Amplified cDNA bands were resolved using 1% agarose TAE gel electrophoresis and detected using the LAS-4000 mini imager (Fujifilm). In addition, four target genes were selected for real-time PCR analysis to confirm their large fold-changes between MDCK and high *siat7e*-expressing MDCK cells. For real-time PCR assays, high capacity cDNA reverse transcription kit (Applied Biosystems) was used to generate cDNA samples. Taqman assays used for gene amplification of *18s rRNA*, *hgf*, *mmp1*, *epcam*, and *tgfβ1* are in this respective order: assay ID 4333760F, assay ID Cf02394141_m1, assay ID Cf02651000_g1, assay ID Cf02647416_g1, and assay ID Cf02623325_m1. Real-time PCR data were processed using the SDS2.3 software (Applied Biosystems). The ΔΔCt method was used to calculate relative fold changes in gene expressions between two samples.

### 4. Western blot confirmations

For the analyses of sialyltransferase 7E and HGF protein expressions, cell pellets were washed twice with cold 1x PBS before being lysed with 1x RIPA buffer (Thermo Scientific) supplemented with complete mini protease inhibitor cocktail (Roche). For the analyses of MET and phosphorylated MET (p-MET) proteins, 1x phosphatase inhibitor cocktail V (Calbiochem) was added to the lysis buffer to preserve phosphorylation on total protein. Rabbit polyclonal antibodies against human sialyltransferase 7E were purchased from Santa Cruz Biotechnology (Cat. No. sc-67348). Goat polyclonal antibodies against human HGF were purchased from Abcam (Cat. No. ab10679). Mouse monoclonal antibodies against human MET and rabbit monoclonal antibodies targeting Tyr1234/1235 phosphorylation sites of MET were purchased from Cell Signaling (Cat. No. 3127 and 3077S, respectively). Total protein contents of all samples were quantified using the BCA protein assay (Thermo Scientific) and normalized accordingly. Samples were run in a 4–12% Bis-Tris gel under reducing conditions at 200 V for 55 min. Gels were blotted against nitrocellulose membranes using the iBlot dry blotting system (Invitrogen). Membranes were blocked using Blocker A/B buffers (Invitrogen) before hybridization with primary antibodies at recommended concentrations. Secondary antibodies conjugated with HRP were used to capture primary antibodies bound on the membrane. After secondary antibodies hybridization, the SuperSignal west pico chemiluminiscent substrate kit (Thermo Scientific) was used to develop the membranes. Protein bands were captured using the LAS-4000 mini imager (Fujifilm) at standard resolution and auto exposure.

### 5. Inhibition of MET phosphorylation in MDCK-*siat7e* cells

PF-02341066 is a proprietary small-molecular inhibitor of MET tyrosine kinase receptor. It was initially identified by Zou et, al [33,39] and then further characterized by Pfizer [32]. It has been reported to have a 50%-inhibition concentration (IC_50_) of 5–20 nM. This drug was a gift from the Center of Cancer Research at the National Cancer Institute (Bethesda, MD). To investigate the role of MET activation in MDCK-*siat7e* cells, these cells were treated with 50 nM of PF-02341066 for duration of 72 h and then withdrawn from the treatment for 96 h. The concentration used is 2x higher than the reported maximum IC_50_ concentration to ensure inhibition of all MET receptors. Light microscopy images were captured and RNA samples were collected every 24 h. The collected samples were then analyzed for *mmp1* gene expression pattern using the same Taqman assay described above.

### 6. ELISA assays on MDCK-*mmp1* clones and measurement of cellular adhesion using shear flow chambers

Stable MDCK-*mmp1* colonies were analyzed for MMP1 secretion levels using a human MMP1 ELISA kit (RayBio). Media collected from various MDCK-*mmp1* colonies were assayed directly in the plate following manufacturer’s protocol. Briefly, 100 μL of standard or sample were added to each well and incubated at room temperature for 2 ½ h. Wells were washed and hybridized with the provided antibody solution. After 1 h incubation, wells were washed again and then developed using HRP-streptavidin and TMB substrate solution. OD values were recorded at 450 nm. Three MDCK-*mmp1* colonies of varying secretion levels and a mock-transfected MDCK colony were selected for analysis of cellular adhesion to tissue-culture treated plates. Three hours before the shear chamber assay, cells were seeded at 4 x 10^5^ cells per dish in 35mm x 10 mm cell culture dishes. A total of 15 dishes were prepared for each subclone. After visually verifying the adhesion of cells to the surface, cells were washed with Hanks’-based cell dissociation buffer (Gibco) for 5 min at 37°C. After washing, cells were subjected to shear stress at 16 and 24 dyn/cm^2^ using the washing buffer as the moving fluid. The numbers of cells within the image frame were counted before and after exertion of stress using the ImageJ software. For each sub clone and each shear stress level, triplicates of samples were assayed.

### 7. Sialidase treatment on MDCK-*siat7e* cells

MDCK-*siat7e* cells were plated at 0.7 x 10^6^ cells/well and treated with 0, 1, and 3 U/mL of sialidase (Sigma-Aldrich) for 30 minutes at 37°C. Protein samples were then prepared from these wells and assayed using Western blots against p-MET and MET to determine the impact on the phosphorylation activity of MET receptors after sialic acid cleavage.

### 8. Pathway analysis and proposed mechanism

The list of differentially expressed genes between parental MDCK and engineered MDCK-*siat7e* cells were imported into NCBI’s publicly available bioinformatics tool, DAVID, for pathway analysis [40,41]. Using DAVID, genes were classified by their functional annotations and then mapped onto the KEGG pathway database. The proposed schematics shown in the following results section was constructed based on the output obtained from pathways in cancer. Additionally, data mining in cBioportal [42,43] was performed to understand the role of ST6GALNAC5 in triggering metastatic transformation in other cell types.

## Results

### 1. Identification of potential target genes differentially expressed in parental anchorage-dependent MDCK and anchorage-independent MDCK-*siat7e* cells

**Fig 1:**
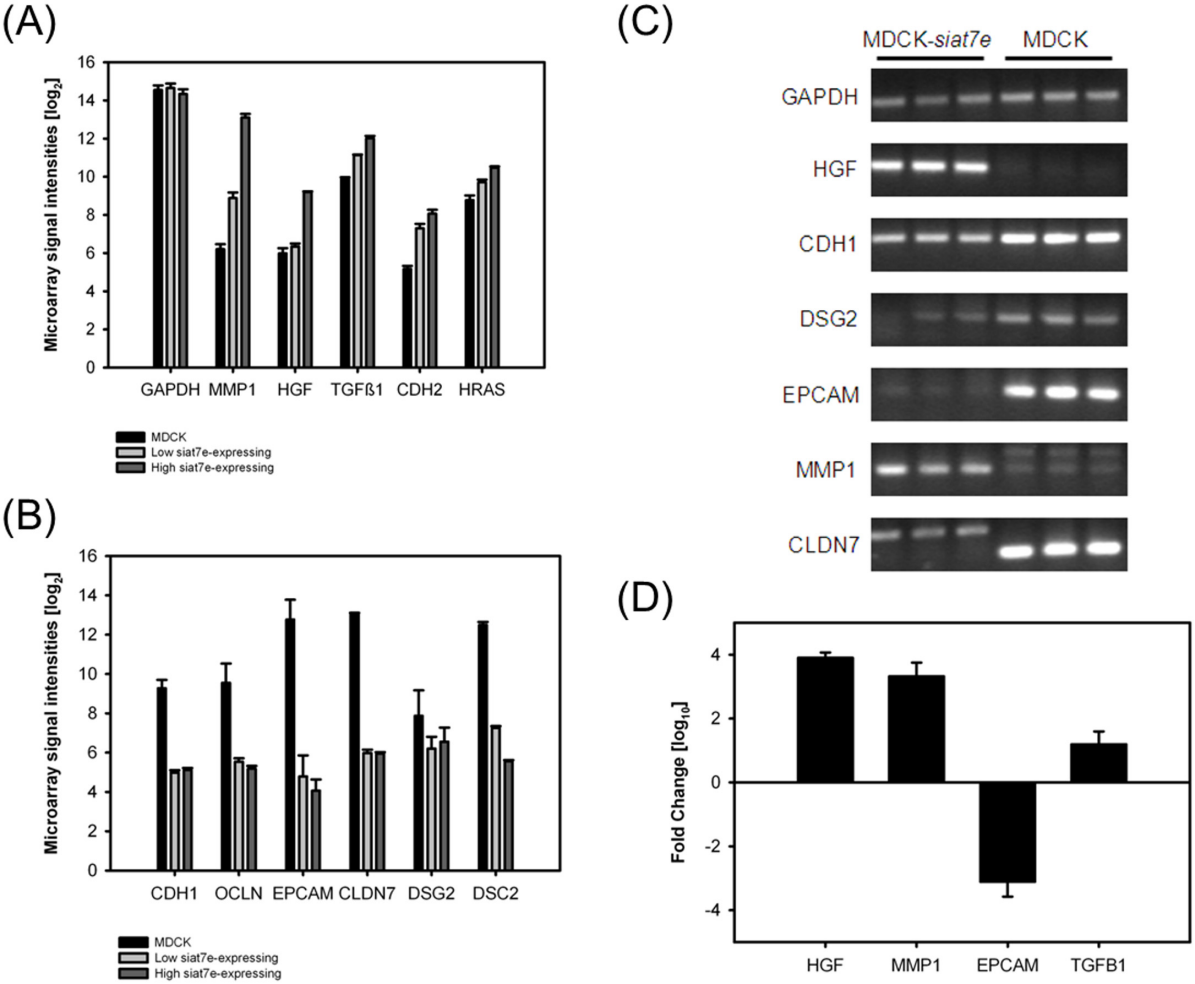
Genes identified from DNA microarray analysis with similar expression trends as the *siat7e* gene. (A) Highly up-regulated genes in MDCK-*siat7e* cells. (B) Highly down-regulated genes in MDCK-*siat7e* cells. (C) End-point PCR confirmation on selected genes using biological replicate samples. (D) Taqman assay results of selected genes (normalized against parental MDCK data).

DNA microarray data from three cell lines (parental MDCK, high *siat7e*-expressing MDCK, and low *siat7e*-expressing MDCK) were processed under stringent conditions to eliminate non-differentially expressed genes from further analysis. The differentially expressed gene list of approximately 700 genes from MDCK and high *siat7e*-expressing MDCK cells can be found in S1 Table. Further selection was done based on biological relevance to cellular adhesion and metastatic transformation, and on correlating trend to the expression level of *siat7e* among the three cell lines. With the inclusion of data from low-*siat7e* expressing MDCK cells, we were able to reduce the large gene list to focus on 11 genes; and the log_2_ values of their microarray signal intensities, compared with the parental MDCK cells, are shown in Fig 1. Several genes were up-regulated in the transformed cells: *mmp1* and *hgf* were over-expressed by more than 10-fold in the high *siat7e*-expressing MDCK cell line (Fig 1A). On the other hand, several genes that are involved in cell-cell adhesion, such as E-cadherin (*cdh1*), occludin (*ocln*), and claudin (*cldn7*), were significantly down-regulated in both MDCK-*siat7e* cell lines (Fig 1B). These results are in agreement with previously reported morphological changes after *siat7e* transfection [7,9]. Since the primary objective was to study the criticality genes responsible for suspension growth, PCR confirmation was performed using parental adherent MDCK cells and fully anchorage-independent MDCK cells expressing higher level of *siat7e*ng. The MDCK cells expressing lower level of *siat7e*, which were less adherent compared to parental MDCK but not fully in suspension, were not included in additional experiments from this point on. As shown in Fig 1C and 1D, differences in mRNA expression were confirmed in six genes by end-point PCR analysis and of those, four were selected for quantitative Taqman assays. After PCR confirmation, *hgf* and *mmp1* were selected for further investigation.

### 2. Overexpression of hepatocyte growth factor in MDCK-*siat7e* cells

**Fig 2:**
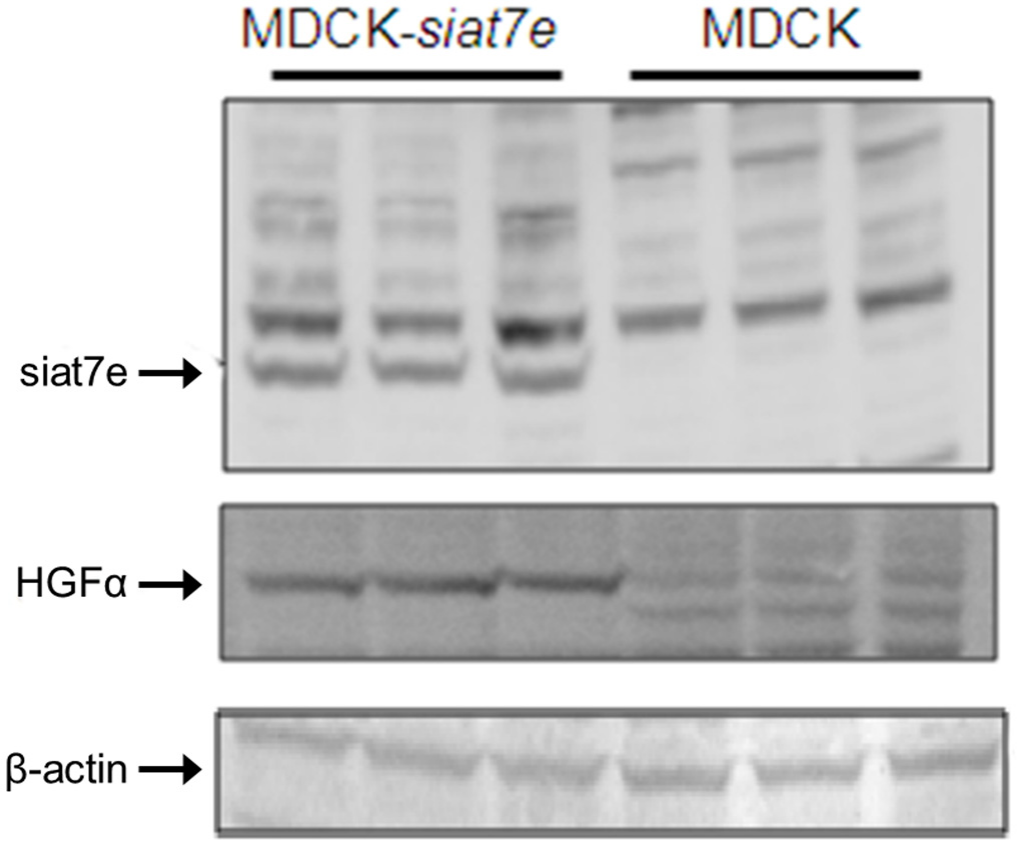
Immunoblot analysis of whole cell lysates of parental MDCK and MDCK siate7e cells, detecting expression of SIAT7E protein, hepatocyte growth factor alpha (HGFα) and Beta-actin expression as loading control. Three biological replicates are shown for both MDCK-*siat7e* and parental MDCK cells.

In addition to PCR confirmation, the presence of HGFα subunit was evaluated by immunoblot to demonstrate differential expression at the protein level. Representative blots of the cell lysate in three biological replicates are shown in Fig 2 indicating the transgene expression of sialyltransferase 7E protein in the engineered MDCK-*siat7e* cells and the expression of HGFα protein in MDCK-*siat7e* cells. Beta-actin expression was also evaluated as a control of constitutively expressed protein. Clearly, higher HGFα protein expression was detected only in MDCK-*siat7e* cells as seen in the first three lanes of the blot. With higher expression of the α subunit, we inferred higher expression of the full heterodimeric HGF protein in anchorage-independent MDCK-*siat7e* cells.

### 3. MET phosphorylation and its impact on the cellular morphology of MDCK-*siat7e* cells

The observation that HGFα was over-expressed in MDCK-*siat7e* cells compared with its expression in the parental MDCK cells hinted towards the connection of the HGF protein with the membrane receptor MET. MET is normally expressed by cells of epithelial origin and HGF is its only known ligand. Following HGF binding, MET’s tyrosine residues in positions 1234 and 1235 become phosphorylated and activated the receptor kinase. The activation subsequently initiated certain cellular programs, including Ras, PI3K, and STAT pathways [27,44].

**Fig 3:**
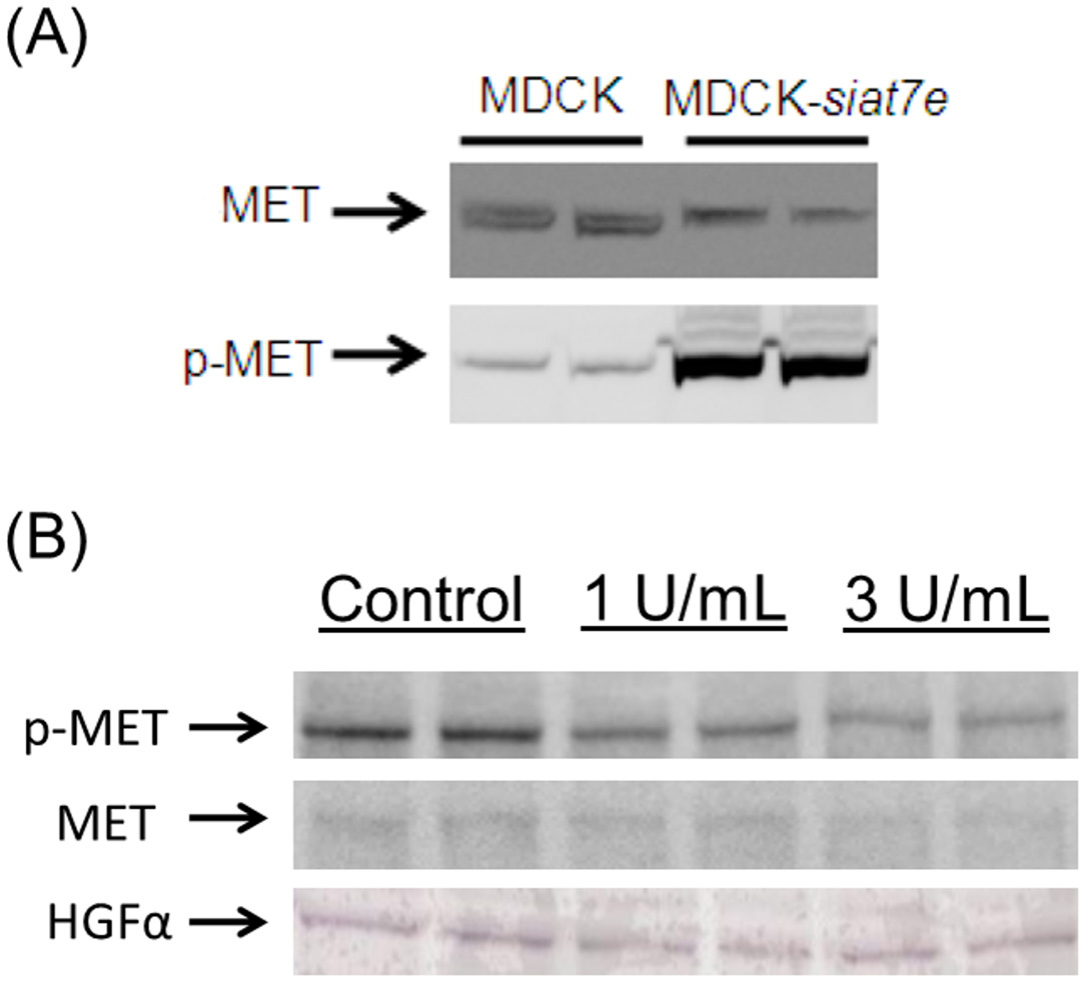
Activation of MET tyrosine kinase receptor in MDCK-*siat7e* cells. Duplicates are shown for each cell line/condition. (A) Cell line comparison of MET and p-MET at Tyr1234/1235. (B) Effect of sialidase treatment on MET phosphorylation in MDCK-*siat7e* cells.

The results of measuring the relative concentration of non-phosphorylated MET and phosphorylated MET (p-MET), by using anti-MET antibodies and anti-Tyr1234/Try1235 MET antibodies, are shown in Fig 3A. The phosphorylation level is significantly higher in MDCK-*siat7e* cells while the expression of non-phosphorylated MET is relatively consistent between the two cell lines. This is in agreement with the observation of elevated levels of both *hgf* mRNA and HGF protein in MDCK-*siat7e* cells.

Treatment of MDCK-*siat7e* cells with exogenous sialidase affected the phosphorylation activities of the MET receptor as shown in Fig 3B. When the phosphorylation activities of the MET receptor were measured by using antibodies targeted specifically against the p-MET, its signal was lowered as the sialidase concentration was increased. This result suggests the dependency of MET activation on the extent of cell surface sialylation.

### 4. Reversal of morphology in the MDCK-*siat7e* cells through MET inhibition

**Fig 4:**
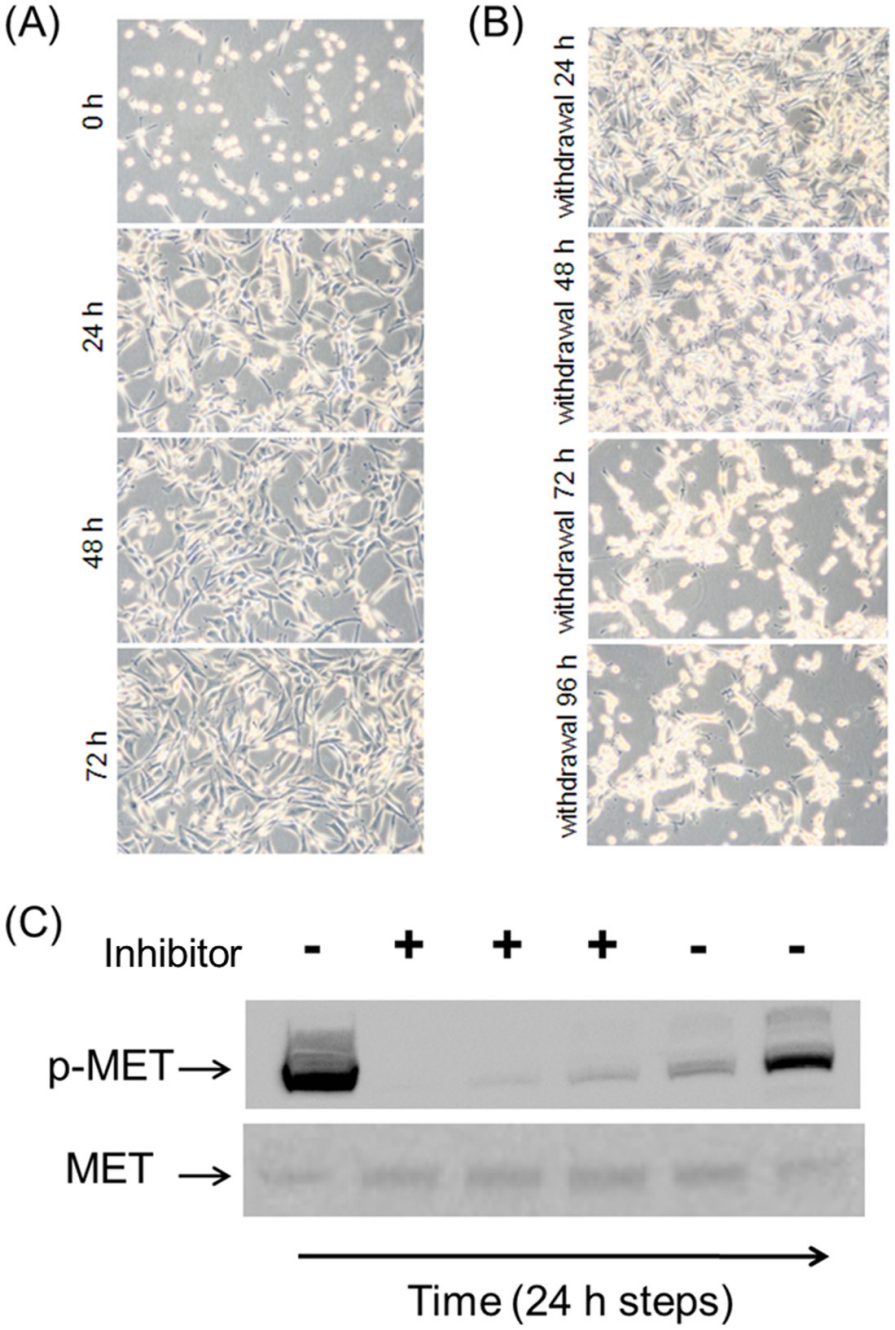
Effect of MET inhibition on cellular phenotypes. (A) Cell morphologies with PF-02341066 treatment and (B) after drug removal from the culture media. (C) Confirmation of the drug’s mechanism of action to inhibit MET phosphorylation status.

To further investigate the role of MET activation in the anchorage-independent MDCK-*siat7e* cells, the cells were treated with PF-02341066, a small-molecular drug that specifically inhibits MET activity [32,33]. The results of treating the anchorage-independent cells with 2.5 times the reported IC_50_ concentration (to ensure all MET activities were subdued) are shown in Fig 4A. Transformations of cell morphology were observed as early as 24 hours after addition of the drug. At 48 hours the cellular phenotype (cell spreading and tight-junction formation) was similar to those of the parental MDCK cells. After 72 h of MET inhibition, the drug was removed from the cells by repeated washing. Consequently, the original morphology was restored at 72–96 h post-withdrawal (Fig 4B). Phosphorylation analysis of MET protein also confirmed the de-phosphorylated state of MET during drug treatment (Fig 4C).

### 5. Over-expression of MMP1 in parental MDCK cells reduces cellular adhesion

**Fig 5:**
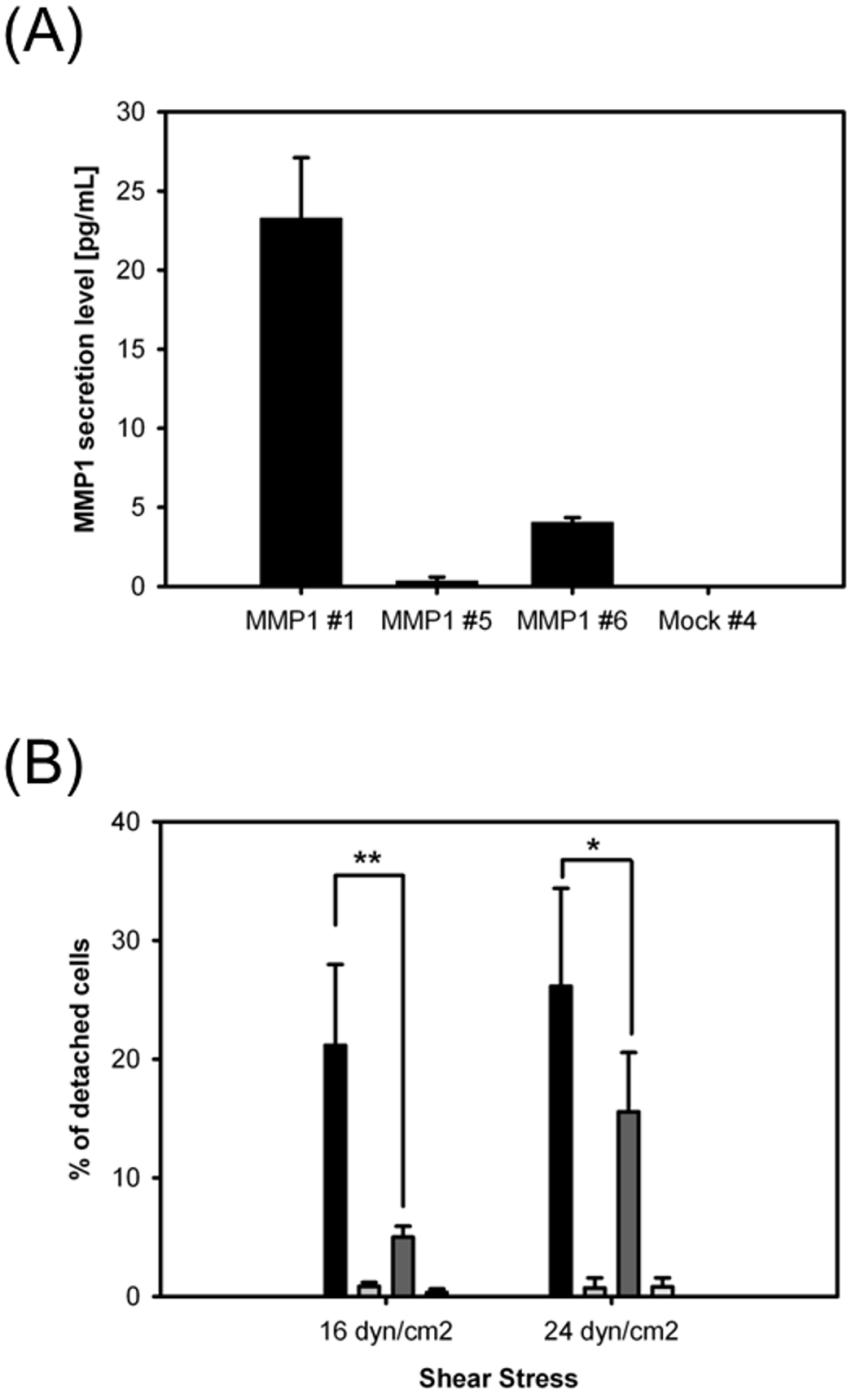
Effect of MMP1 secretion on cellular adhesion in MDCK cells. (A) MMP1 secretion level of stable MDCK-*MMP1* colonies. (B) Cellular adhesion quantified by shear flow chamber assays. Significance: ** p-value < 0.03, * p-value > 0.05.

In addition to *hgf*, *mmp1* was also over-expressed in MDCK-*siat7e* cells (Fig 1). Transcript levels of *mmp1* increased in parental MDCK cells in response to treatment with exogenous, recombinant HGF protein (data not shown). To evaluate the effect of this gene on the cellular adhesion of the parental MDCK cells, an expression vector containing the human *mmp1* gene was constructed and stable MDCK cell lines expressing *mmp1* were isolated. Fig 5A shows the amount of protein secreted from the isolated clones as determined by ELISA. Three sub-clones with varying levels of secreted MMP1 enzyme (in the range of 0.3 pg/mL to 23 pg/mL) and a mock-transfected clone were selected for the shear flow chamber assay. At the force of 16 dyn/cm^2^, the percentage of detached cells correlated with the MMP1 secretion levels (Fig 5B). Approximately 21% of total cells were detached in the MMP1 #1 sub-clone and less than 1% in MMP1 #5 sub-clone and mock-transfected sub-clone. Although, the correlation became weaker between the top two MMP1-expressing sub-clones when the shear stress force was increased to 24 dyn/cm^2^, the percentage of detached cells in MMP1 #6 and mock-transfected sub-clones remained less than 1% of the total cell population.

### 6. Proposed pathway for the modified phenotype

**Fig 6:**
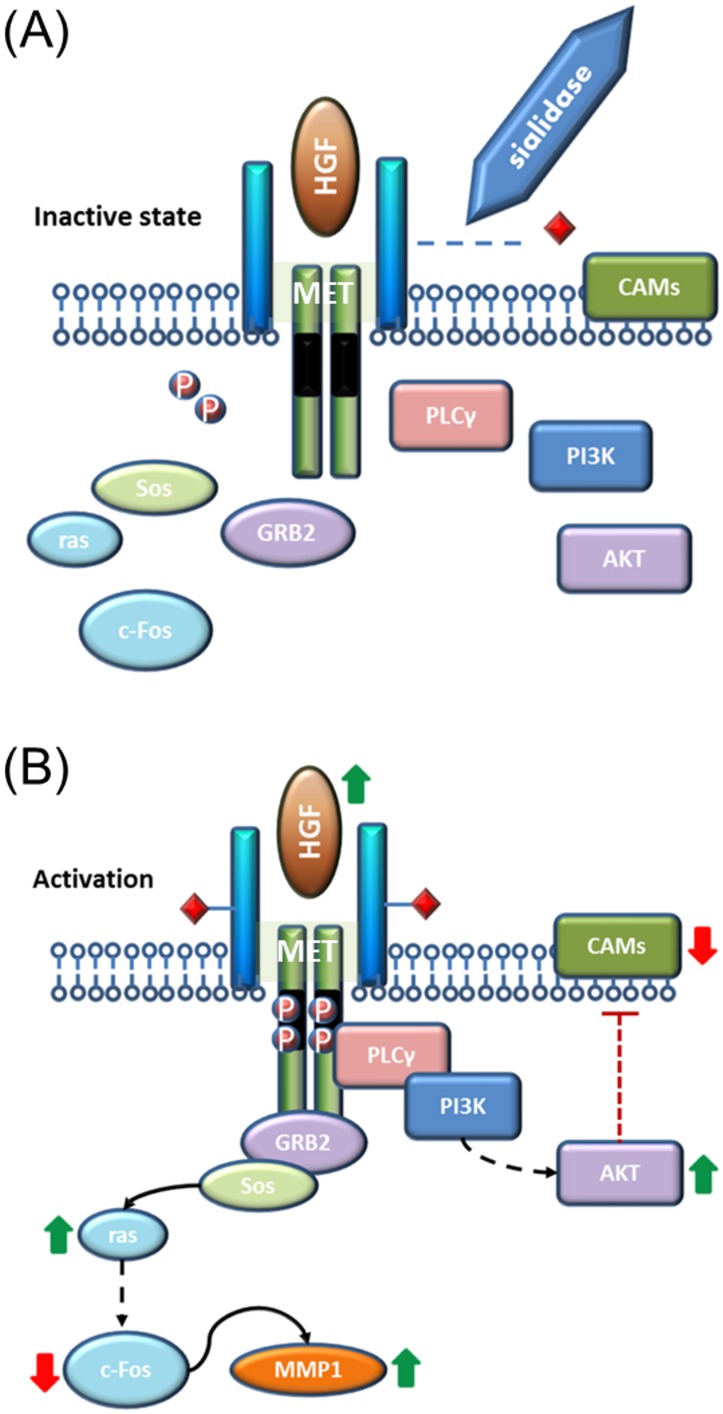
Proposed mechanism of MET sialylation affecting the activation of signaling cascades to disrupt anchorage-dependence. Diagrams are simplified based on known mechanisms. (A) Hyposialyation leads to diminished response of the receptor and thus no pathway activation. (B) Sufficient sialylation on MET allows activation by its ligand, HGF, causing up-regulation of MMP1 and down-regulation of cell adhesion molecules.

Based on the microarray results summarized in the S1 Table, analysis using the NCBI’s DAVID bioinformatics tool was performed. All differentially expressed genes were clustered by functional annotation, and approximately half were mapped onto a number of pathways within the KEGG database, including cell adhesion molecules and pathways in cancer (see S2 and S3 Tables). The pathway mapping analysis using the DAVID bioinformatics tool showed up-regulation of multiple genes along the PI3K/AKT and RAS signaling pathways, which are directly linked to MET activation upon ligand binding. A significant up-regulation of the *mmp1* transcript level can be traced back to the increased *ras* transcription activity, despite the microarray observation of slight down-regulation of *c-Fos*, the transcription factor known to induce *mmp1* gene expression. Up-regulation of *akt* was also observed. Based on the pathway analysis and the sialidase experimental results, a proposed mechanism linking sialylation of the MET receptor to its downstream pathways is described in Fig 6. The result of MET phosphorylation leads to up-regulation of *mmp1* and initiation of PI3K/AKT pathways to down-regulate cell adhesion molecules. We also propose that hyposialylation would dampen the phosphorylation level of the MET receptor and, therefore, result in the disengagement of its downstream pathways.

## Discussion

Previous reports on the cellular morphology and growth characteristics of MDCK cells showed that as a result of stable transfection with the human *siat7e* gene, the engineered MDCK-*siat7e* cells acquired different growth characteristics and were able to grow in suspension [8,9]. We hypothesized that this major transformation may be the result of activating one or more signaling cascades caused by the increase in α2,6-linked sialylation. To address the question of whether any major pathways were activated in the transformed MDCK-*siat7e* cells, transcriptome activities of the parental and the transformed cells were profiled using DNA microarrays. Following stringent data reduction (p-values less than 0.01), a small subset of genes with expression levels that correlate well with the expression of *siat7e* in MDCK cells were selected for further investigation. The expression levels of the selected genes (Figs 2 and 3) indicated a significant match between the transcript activities in *siat7e*-expressing cells and markers commonly observed during EMT [24]. Two of the markers These markers are the down-regulation of *cdh1*, the gene encoding E-cadherin, and the up-regulation of *cdh2*, the gene encoding N-cadherin, in the *siat7e*-expressing lines. These findings suggest that the loss of cell-cell adhesion and the ability to proliferate in suspension are the results of an activated EMT process. The significant over-expression of HGF in the MDCK-*siat7e* cells, detected by both mRNA and protein expression, supports the hypothesis of EMT activation (Figs 1 and 2). Interestingly, a recent study in human breast adenocarcinoma (MDA-MB-231) cells has shown that silencing ST6GALNAC5 using miRNA led to increased expression of epithelial cell adhesion molecules and the reversal of the EMT process [45]. In that particular study, both transient transfection of miR-200b (verified to target ST6GALNAC5) and ST6GALNAC5 knockout led to higher E-cadherin expression and visible change in morphology of the MDA-MB-231 cells. The authors also measured ST6GALNAC5 expression in another human lung carcinoma cell line (A549) after inducing EMT by TGF-β1 treatment and observed its increase at the protein level. These results from two different cell lines implicate the role of ST6GALNAC5 within the EMT-signaling network. In addition, data-mining using cBioPortal showed significant amplification and mutation of ST6GALNAC5 in at least 14 independent studies (see S1 Fig). Taken together, these data suggest that the reported findings could be relevant to other cell lines as well.

The ability of HGF to initiate EMT through the activation of the MET receptor is well documented [46,47], although conflicting results have been reported [48,49]. The MDCK-*siat7e* cells were able to produce endogenous HGF and to activate EMT in an autocrine fashion unlike the classic paracrine action of HGF-mediated EMT. Prior to the current study, similar observations of autocrine activation of the MET receptor in MDCK cells, mouse myoblast cells, and bovine ovarian surface epithelium cells have been reported [50–52]. It has also been shown that switching from the paracrine mechanism to an autocrine loop in the activation of MET is feasible [53]. Since the *siat7e*-expressing MDCK cells are able to produce their own MET ligand, HGF, the question of whether the binding of HGF to MET can occur within the cytoplasm and, more critically, the functionality of non-membrane bound HGF: MET complex are issues which should be addressed, but were beyond the scope of this study.

The role of the MET activation in transforming the phenotypic behaviors of the MDCK-*siat7e* cells was confirmed using the specific MET small-molecule inhibitor [32,33]. At a concentration more than twice the reported IC_50_, visible changes of cell spreading, cell-cell adhesion, and cell-matrix adhesion in MDCK-*siat7e* cells were observed as early as 24–48 h (Fig 4). Drug withdrawal after 72 h of MET inhibition led to gradual reversal of the morphology, although cell clumping was still present after 96 h. The time-scale requirement for the transformations suggests that complex signaling cascades may be involved downstream of the MET receptor activation. These results establish the significance of the MET receptor tyrosine kinase in its ability to direct the transformation of the MDCK cells following transgenic *siat7e* expression. One of the highly up-regulated genes in the suspension growing MDCK cells was *mmp1*. This was found to be in agreement with a prior study reporting on an unusual up-regulation of mmp1 transcription in EMT-induced MDCK cells through *ras* transformation [36], and in our observation of increased *mmp1* expression in parental MDCK cells under HGF stimulation (data not shown). Based on these observations, the human *mmp1* cDNA was cloned and stably expressed in MDCK cells to study its impact on cell attachment. By using shear chamber assays, the cell-matrix adhesion properties of various MDCK-*mmp1* sub-clones were quantified and were shown to correlate with the secreted levels of MMP1 (Fig 5). Given the known functionalities of matrix metalloproteinase enzymes and their associations in metastatic transformations [37, 54], it is likely that in addition to the MET activation; MMP1 plays a role in affecting the anchorage-independence of the MDCK-*siat7e* cells.

The challenge is to understand the connection between over-expression of sialyltransferase and the activation of an autocrine HGF/MET loop in the MDCK cells, since this enzyme can affect many substrates within the cell. Reports of the effects of sialylated compounds on invasive growth, cell adhesion, and activation of signaling kinases are prevalent [17,55–58]. In vitro stimulation of endogenous HGF production by the addition of sialylated glycolipids have also been demonstrated in human glioma cells [59]. Stimulation of endogenous HGF expression could be occurring in the MDCK cells as a result of an increased α2,6-linked sialic acid composition. On the other hand, the MET receptor is known to be a highly sialylated structure. Thus it is also possible that *siat7e* expression can influence the phosphorylation state of the MET receptor and, consequently, activate the signaling cascades. As a result of using sialidase treatment (Fig 3), we showed that there is a link between sialylation and MET activation but the exact mechanism is still unclear. Recently a study demonstrated the activation of the MET pathway through ST3GAL4 expression [60]. The investigator also provided supporting evidence that a sialylated glycan structure is a requirement for MET phosphorylation. In another study, it was shown that aglycosylated HGF was still capable of triggering the cell scattering phenomenon in MDCK cells [61]. This would suggest that the effect we observed with sialidase treatment was not likely due to glycan changes on the ligand. Also, it was demonstrated elsewhere that hyposialylation of MET can lead to changes in cell motility [62]. Based on the collective findings described in this work, we propose that activation of the MET receptor stimulates *ras* expression which eventually causes up-regulation of matrix metalloproteinases, including *mmp1* [36,63], and also initiates the PI3K and AKT pathways to down-regulate cell-adhesion molecules [63,64]. We propose that sialic acid residues on MET are essential for its phosphorylation and activation, perhaps due to conformational changes (Fig 6). Further studies are required to explain the mechanism of MET’s dependency on sialylation for phosphorylation. The engineered MDCK-*siat7e* cells would serve as a good model for EMT signaling analysis to investigate the requirements for MET activation.

## Supporting Information

S1 Fig Data-mining in cBioPortal database. Search results in the cancer genomics database indicated at least 14 independent studies in various cancer cell lines showed significant amplification and/or mutation of the ST6GALNAC5 gene.
(PDF)

S1 Table List of the differentially expressed genes. Approximately 700 gene were detected as differentially expressed genes between parental MDCK and MDCK-*siat7e* cell lines.
(XLSX)

S2 Table Functional clustering of genes. Differentially expressed genes were clustered into 46 groups based on their functional gene annotations.
(XLS)

S3 Table Mapping of genes onto various pathways. Differentially expressed genes were mapped onto various pathways from the KEGG database.
(XLS)
