# Supplementary figures and images for: Stable Ectopic Expression of ST6GALNAC5 Induces Autocrine MET Activation and Anchorage-Independence in MDCK Cells
Source: PLoS One. 2016 Feb 5;11(2):e0148075. doi: 10.1371/journal.pone.0148075 (PMC4743853; doi:10.1371/journal.pone.0148075)

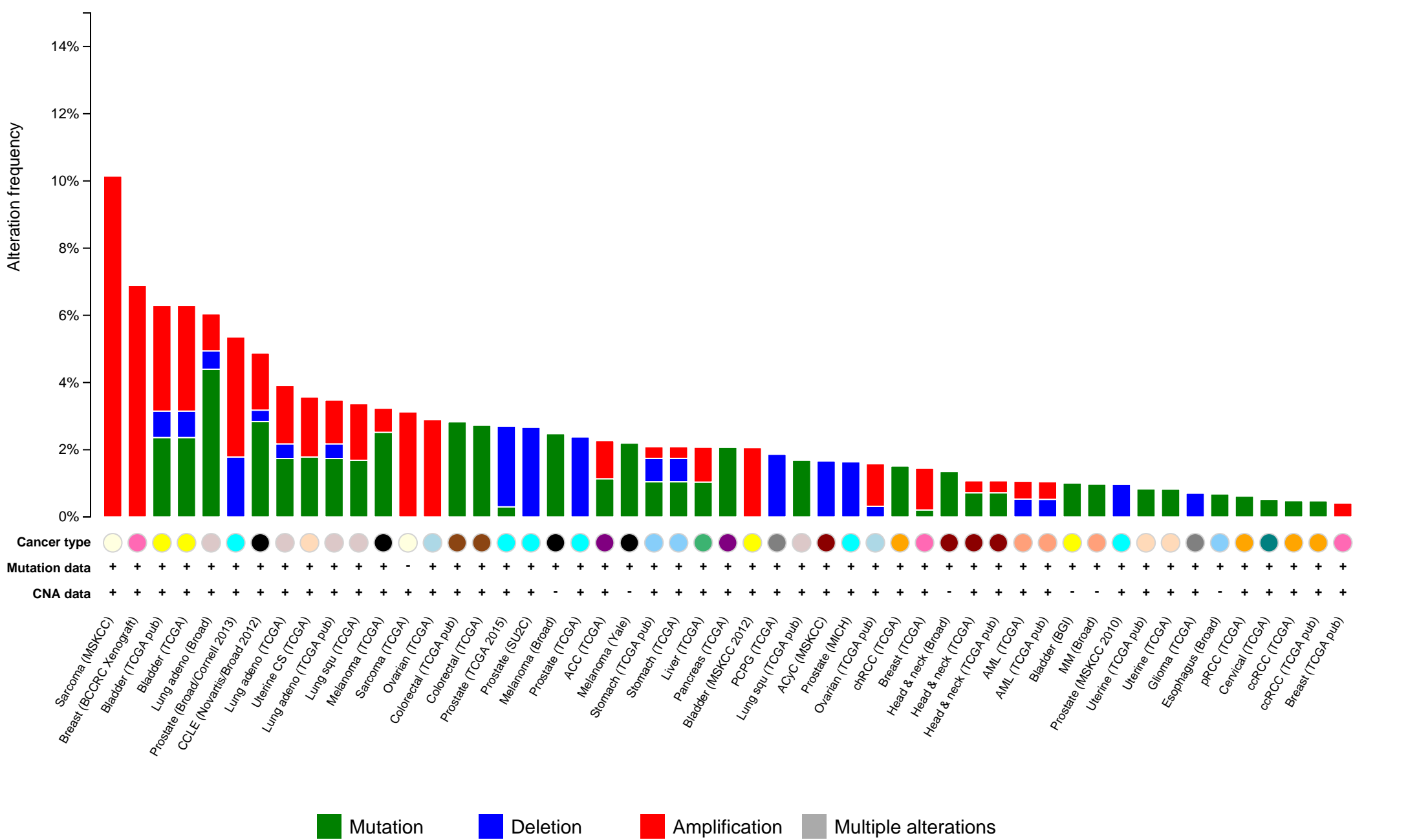

Supplement: S1 Fig — Search results in the cancer genomics database indicated at least 14 independent studies in various cancer cell lines showed significant amplification and/or mutation of the ST6GALNAC5 gene. (PDF) [file pone.0148075.s001.pdf]
